# Estimation of the Exposure of the UK Population to the Bovine Spongiform Encephalopathy Agent through Dietary Intake During the Period 1980 to 1996

**DOI:** 10.1371/journal.pone.0094020

**Published:** 2014-04-15

**Authors:** Chu-Chih Chen, Yin-Han Wang

**Affiliations:** Division of Biostatistics and Bioinformatics, Institute of Population Health Sciences, National Health Research Institutes, Zhunan, Taiwan; University of Verona, Italy

## Abstract

Although the incidence of variant Creutzfeldt-Jakob disease (vCJD) has declined to 1 since 2012 in the UK, uncertainty remains regarding possible future cases and the size of the subclinical population that may cause secondary transmission of the disease through blood transfusion. Estimating the number of individuals who were exposed to the bovine spongiform encephalopathy (BSE) infectious agent and may be susceptible to vCJD will help to clarify related public health concerns and plan strategies. In this paper, we explore this estimate by describing the probability of potential exposure due to dietary intake throughout the BSE epidemic period from 1980 to 1996 as a stochastic Poisson process. We estimate the age- and gender-specific exposure intensities in food categories of beef and beef-containing dishes, burgers and kebabs, pies, and sausages, separating the two periods of 1980–1989 and 1990–1996 due to the specified bovine offal legislation of 1989. The estimated total number of (living) exposed individuals during each period is 5,089,027 (95% confidence interval [CI] 4,514,963–6,410,317), which was obtained by multiplying the population size of different birth cohorts by the probability of exposure via dietary intake and the probability of survival until the end of 2013. The estimated number is approximately doubled, assuming a contamination rate of 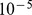. Among those individuals estimated, 31,855 (95% CI 26,849–42,541) are susceptible to infection. We also examined the threshold hypothesis by fitting an extreme-value distribution to the estimated infectious dose of the exposed individuals and obtained a threshold estimate of 13.7 bID_50_ (95% CI 6.6–26.2 bID_50_) (Weibull). The results provide useful information on potential carriers of prion disease who may pose a threat of infection via blood transfusion and thus provide insight into the likelihood of new incidents of vCJD occurring in the future.

## Introduction

The incidence of variant Creutzfeldt-Jakob disease (vCJD) in the UK has declined to very few cases in recent years [1], and there is no evidence that the tail in the epidemic will be self-sustaining [2]. However, great uncertainty remains as to the number of asymptotic or subclinical infected individuals in the population. This uncertainty is of serious concern, especially with regard to the potential risk of human-to-human secondary transmission via blood transfusion, plasma products, or through contaminated surgical instruments [3]–[7].

Several large-scale studies have been performed to estimate the prevalence of incubating individuals or carriers of the infectious agent by screening lymphoreticular tissues of appendix or tonsil specimens removed during routine surgery [8]–[13]. However, these results are inconclusive, with outcomes ranging from negative [9] to 1 in 10,000 [6], [7], [10], [12] and 1 in 4,000 [6], [7], [11]. Recently, a new large-scale survey showed that the prevalence of abnormal prion protein in the appendix is even higher, with it being present in approximately 1 in 2000 [13]. Noticeably, based on the study outcomes, the prevalence of abnormal prion protein does not differ among various birth cohorts and between genders, and it is even higher for the valine homozygous (VV) genotype at PRNP codon 129 than for the methionine homozygous genotype (MM). The recent development of a blood test to detect vCJD may offer a new way to identify infected individuals [12], [14]. However, the screening test is not yet fully in place, and further confirmative tests will be needed to address the issue of sensitivity and specificity [12].

To estimate the number of infected individuals who may develop into a vCJD case, mathematical models, such as the back-calculation technique originally developed in the context of the HIV/AIDS epidemic, have been widely applied [15]–[22]. Although this methodology has been successful in predicting the vCJD epidemic, it is noted that predictions based on the observed vCJD cases are unable to indicate the prevalence of infection (or subclinical carriers) in the population [21], [23]. Alternative simulation-based methods using dietary exposure to BSE infectious agents among different birth cohorts have also been proposed to predict future incidence of vCJD [24]–[27]. In addition to the results on age-dependent consumption of meat products from the British dietary surveys [28], [29], Cooper and Bird estimated the intensities of dietary exposure to bovine spongiform encephalopathy (BSE) through the consumption of mechanically recovered meat (MRM) and head meat in burgers, sausages, and other meat products by birth cohort and gender [30]–[32]. The researchers then estimated the number of infections in calendar year *y* for each birth cohort by assuming proportionality to the birth cohort’s dietary exposure to BSE in that year and found the best fit based on a goodness-of-fit criterion [25]. Other mathematical modeling methods include using hidden Markov models [33] considering state transitions or a stochastic model for vCJD transmission that takes into account known transmission routes [2]. However, the aim of these methods is to predict future vCJD cases, rather than to estimate the prevalence of the subclinical population.

The study’s primary goal is to estimate the number of potential carriers that might pose a threat of secondary transmission of the prion disease through the estimated number of exposed individuals by different age groups during the period 1980–1996 in the UK. Additionally, we further justify the threshold hypothesis [4], [34]–[39] and provide a threshold estimate by comparing the observed incidence of vCJD cases. We estimate the number of exposed individuals by multiplying the population size of different birth cohorts in the UK [40] by their corresponding probability of exposure during the BSE epidemic period 1980–1996. The exposure probability and the number of repeated dietary exposures to a BSE infectious agent is described by a Poisson process, taking into account the probability that an individual may have consumed an infectious agent more than once during the period [41]. For different meat products – beef and beef-containing dishes, burgers and kebabs, pies, and sausages – we calculate the exposure intensity for the Poisson process using the ratio of the estimated amount of BSE infectious agent to the total consumption for the two major periods 1980–1989 and 1990–1996, separately. Two estimates are made: one without contamination and another considering a contamination rate (CR) of 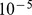 in the production of MRM containing the BSE infectious agent. We then multiply the estimated number of exposed individuals in each birth cohort by the corresponding posterior estimate of age-specific susceptibility [13], [17] using a Bayesian statistic approach and the survival probability [40] to obtain the number of susceptible exposed individuals who were alive at the end of 2013. These individuals are most likely the subclinical subpopulation, but they may not develop into a vCJD case if they were exposed to a low infectious dose [4], [36]–[39]. To further justify the threshold hypothesis, which states that an individual must be exposed to a very high dose to trigger the exponential growth of abnormal prions in the brain [35], we fit an extreme-value distribution to the susceptible exposed individuals for their exposure dose. An estimate of the threshold dose is obtained by comparing the model-fitting outcomes with the observed vCJD cases in each birth cohort.

## Materials and Methods

### Data Sources

Dietary exposure to the infectious agent occurs mainly through the specified risk material (SRM) of the BSE-infected cattle slaughtered for food consumption. Two primary periods of BSE infection may have existed during the 1980–1996 period in the UK. The first period is between 1980 and the specified bovine offals (SBO) legislation of November 1989; the second period is between the SBO ban and the Over Thirty Month Rule (OTMR) of March 1996 [42]. After the introduction of the OTMR in 1996, the BSE epidemic is considered to have been under effective control, and dietary exposures to BSE-infected animals slaughtered for consumption are negligible thereafter [18], [43]. Due to the effects of these legislations, we mainly divide the population cohorts and the intensities of BSE infectious agent from dietary exposure into two periods: 1980–1989 and 1990–1996.

For exposure to the BSE infectious agent during the two periods, we counted only those cattle in the last year of incubation and unreported cases or slaughtered cattle for totals of 42,809 from 1980–1989 and 236,763 from 1990–1996 [16]. The numbers are digitally read using Plot Digitizer 2.6.3 (available at http://plotdigitizer.sourceforge.net) and summarized from Figure 2 (c) of Donnelly et al. [16]. The study based on new biochemical testing doubles the previous estimated epidemic size [15], which was likely seriously underestimated [44].

We estimate the amounts of SRM, including brain, spinal cord, and dorsal root ganglia (DRG), from a BSE-infected bovine separately for the two periods. Table 1 summarizes the estimated amounts of brain, spinal cord, and DRG removed with head meat and the amount of MRM from one bovine carcass during each of the legislation periods [31], [32], [42]. We exclude SRM of the trigeminal ganglia, ileum, tonsil, spleen and eyes because these parts are typically removed before meat consumption [42]. The total amounts of MRM and head meat are digitally read using Plot Digitizer 2.6.3 from Figure 3 of Cooper and Bird [31] and Figure 2 of Cooper and Bird [32], respectively. Among these meat products, we adopt the inclusion rates of MRM and head meat given in the DNV report [45] because of more specific information. For infectivity in consumed MRM and head meat, we adopt the estimate that 1 g of the BSE-infected brain and spinal cord contains 10 bID_50_ [39] and derive the relative infectivity in infected DRG based on Arnold et al.’s estimate [46].

**Table 1:** Estimated amounts of brain material removed with head meat, and spinal cord and DRG from each BSE-infected bovine carcass slaughter for consumption during 1980 to 1996.

Legislative period	Brain^a^	Spinal Cord^b^	DRG^b^
Before November 1989 (SBO ban)	1.65 g (0.4–4.0 g)	3.3 g (0.24–12.02 g)	27 g
After November 1989: November 1989 to May 1990	1.83 g (0.4–5.1 g)	1.5 g (0.02–8.30 g)	27 g
June 1990 to February 1992	1.32 g (0.4–3.1 g)	1.5 g (0.02–8.30 g)	27 g
March 1992 to March 1996 (OTMR)	1.28 g (0.4–2.9 g)	1.5 g (0.02–8.30 g)	27 g

a Based on the information given in Cooper and Bird [32] and Comer and Huntly [42].

b Based on the information given in Cooper and Bird [31].

The SRM infectious agent is mainly contained in MRM and the head meat used in different meat product categories such as burgers, sausages, and other meat products in different proportions during the BSE epidemic period [30]–[32], [42], [45]. Therefore, we calculate the exposure intensities for the four main bovine meat products: beef and beef-containing dishes, burgers and kebabs, pies, and sausages separately based on National Diet and Nutrition Survey (NDNS) consumption data [29]. We assess dietary exposure according to the percentages of consumers and age- and gender-specific daily consumption as reported in the NDNS 2008/2009 (Tables 5.2a to 5.2c) [29]. The category of ‘beef and dishes’ includes beef and veal joints, steaks, minced beef, stewing steak, casseroles, meat balls, lasagna, chili con carne, beef curry, bolognaise sauce, shepherd’s pie, and canned beef. ‘Burgers and kebabs’ includes beef burgers, hamburgers, cheeseburgers, doner/shish/kofte kebabs, grill steaks, and steaklets. ‘Sausages’ includes beef, pork, turkey sausages, bologna, sausages in batter, saveloy, frankfurters, and sausage dishes. ‘Meat pies and pastries’ includes any type of meat [29].

In order to calculate the exposure intensities for different age groups, the age grouping system of 1–3 years, 4–10, 11–18, and 19 and older (19+) of the NDNS in the UK [29] is adopted. The populations of different birth cohorts and life table information are obtained from UK national statistics [40]. We obtained the year and age at onset of the vCJD cases data from the UK Creutzfeldt-Jakob Disease Surveillance Unit [1] through personal communication. For the probability of infection after being exposed, we assigned a prior distribution to the susceptibility estimate of Valleron et al. [17] and incorporated the large-scale prevalence survey outcomes of abnormal prion in human appendixes [13] to obtain a posterior estimate. We estimated the number of subclinical carriers of the MM, MV, and VV genotypes at PRNP codon 129 according to their relative proportions of 0.37, 0.51, and 0.12 in the UK population [6], [36].

### Intensity of Infectious Agent in Bovine Meat Products

Because MRM is produced in batches of five to seven tons, the MRM from one infected bovine could contaminate an entire batch [47]. Assuming that at most one infected bovine is contained in one batch of MRM, the number of contaminated batches would be equal to the total number of infected bovines used in producing MRM. Therefore, for a batch weighing six tons, the total amount of contaminated MRM is

*W*  =  Number of one-year pre-clinical bovines used in MRM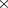 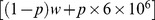, (1)where *w* is the amount of infectious agent in MRM from one infected bovine and *p* is the CR of MRM in the same batch. Suppose that a typical bovine carcass yields approximately 6 kg of MRM [45], then a batch of MRM weighing six tons will need 1000 carcasses. Therefore, with 1% of the contamination on the carcass that is transferred to meat during boning and packing operations [42], the CR will be 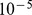 if one carcass is infected with BSE in a batch.

Given the total amount of contaminated MRM, the amount in the *l*-th type of bovine meat products is



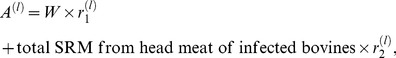
(2)where 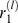 and 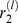 are the proportions of MRM and head meat used in the *l*-th type of bovine meat products: 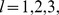 and 4 represent beef and dishes, burgers and kebabs, pies, and sausages, respectively. A percentage of 25% and 41% is used in calculating the proportion of beef in pies and sausages, respectively, based on the 1994 total consumptions of various types of meat in the UK [48], [49]. The total consumption of the *l*-th type of bovine meat products is



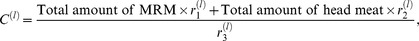
(3)where 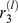 is the proportion of MRM and head meat contained in the *l*-th type of bovine meat products.

Great uncertainties and variations are reported in the literature concerning the proportions of MRM and head meat used in different meat products throughout the 1980–1996 period [30]–[32], [42], [45]. Therefore, we adopt a Bayesian probabilistic approach of Markov chain Monte Carlo (MCMC) simulations using WinBUGS 1.4.3 (MRC Biostatistics Unit, Cambridge, UK) with one thousand runs for the intensities. The mean of the prior distribution represents data from the adopted main reference and the corresponding standard deviation covers uncertainty in the data (including data from other references).

A Dirichlet distribution with appropriate prior parameters is employed to generate 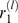, 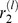 so that the proportions of the four types of bovine meat products is summed up to 1. Also, a beta distribution is employed for the prior distribution of each of the proportion parameters. A beta distribution is employed to generate 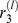. It is estimated that the annual MRM production during the period is 5000 tons, with 2000 tons split into burgers (40%), 2000 tons into frozen mince (40%), and 1000 tons into minor uses including pet food and export (20%) [45]. Therefore, the mean of *r_1_* for burgers is set at 0.41, and the means for beef and dishes and pies are set at 0.19 and 0.20, which are split evenly from the frozen mince. The proportions of head meat in each food category are similarly determined. Table 2 lists the simulation means and standard deviations of the proportions *r_1_*, *r_2_*, and *r_3_* for beef and dishes, burgers and kebabs, pies, and sausages, respectively. Because a certain proportion of MRM and head meat is intended for minor uses (e.g., pet food), the total mean of *r_1_* and *r_2_* in Table 2 are rescaled before and after the simulations for their relative rates across the four food categories. The inclusion rates of MRM and head meat in each food category for *r_3_* to retrospectively estimate the total consumption of the *l*-th type of bovine meat products are obtained mainly based on Table 3.1 and Section 3.2 of the DNV report [45] and the Scientific Steering Committee report [47].

**Table 2:** Means and standard deviations (std) of the proportions *r_1_*, *r_2_*, and *r_3_* used in the intensities.

Proportion^a^	Beef and dishes		Burgers and kebabs		Pies		Sausages	
	mean	std	mean	std	mean	std	mean	std
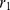	0.19	0.30	0.41	0.40	0.20	0.30	0.03	0.10
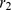	0.19	0.30	0.40	0.30	0.19	0.30	0.03	0.10
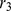	0.40	0.20	0.20	0.12	0.40	0.20	0.03	0.02

a Based on the information given in the 2002 DNV report [45] and the 1999 Science Steering Committee report [47].

**Table 3:** Estimated numbers of exposed individuals and subclinical carriers, the mean exposed dose and the threshold estimates in bID_50_ for vCJD infection [43], and the expected numbers of vCJD cases exceeding the threshold dose based on the Weibull and Frechet extreme-value distribution fittings separately, assuming that the contamination rate is 0.

Birth cohort	Number of exposed individuals^a^		Number of subclinical carriers		Mean exposure dose^b^	Extreme value distribution	Parameter estimates		Threshold dose estimate	Predicted vCJD cases	Observed vCJD cases^c^
		MM	MV	VV							
							α	σ			
Pre-1970	3,581,316	5,260	3,505	7,015	0.73	Weibull	1.3 (1.2–1.4)	2.0 (1.6–2.3)	11.6 (9.5–13.1)	66 (50–82)	65
	(3,176,511–4,496,682)	(4,661–6,609)	(3,106–4,405)	(6,216–8,813)		Frechet	1.5 (1.3–1.7)	0.017 (0.0060–0.031)	10.1 (9.5–10.8)	67 (52–83)	
1970–1979	814,847	2,660	1,771	0	0.64	Weibull	1.8 (1.5–2.3)	3.4 (2.2–4.0)		68 (53–85)	69
	(725,913–1,024,476)	(2,367–3,348)	(1,576–2,230)	(0–0)		Frechet	1.1 (1.0–1.3)	0.007 (0.0022–0.019)		71 (56–88)	
1980–1989	573,099	2,431	0	0	0.44	Weibull	1.2 (1.1–1.4)	2.0 (1.7–2.4)		40 (28–54)	40
	(507,631–732,109)	(2,152–3,111)	(0–0)	(0–0)		Frechet	1.1 (0.8–1.3)	0.006 (0.0003–0.019)		42 (31–56)	
1990–1996	119,765	3,309	4,803	1,101	0.26	Weibull	1.6 (0.7–3.7)	0.7 (0.2–2.0)		0 (0–1)	0
	(104,908–157,050)	(2,461–4,993)	(3,504–7,350)	(806–1,682)		Frechet	1.3 (1.0–1.8)	0.006 (0.0002–0.030)		2 (0–5)	
Total	5,089,027	13,660	10,079	8,116		Weibull				174 (131–222)	174
	(4,514,963–6,410,317)	(11,641–18,061)	(8,186–13,985)	(7,022–10,495)		Frechet				182 (139–232)	

The numbers in parentheses are the corresponding 95% confidence intervals.

a The number of exposed individuals are for all genotypes who were alive at the end of 2013.

b The mean exposure dose is obtained by dividing the estimated ratio of consumption of total amount of infectious dose in bID_50_ for each birth cohort by the corresponding number of exposed individuals who were alive at the end of 1996.

c The three cases infected through blood transfusion are excluded [1].

### Age- and Gender-specific BSE Exposure Intensities and Probabilities during the Period 1980 to 1996

Because the BSE infectivity among meat products may have varied over the course of the BSE epidemic period, especially between the two major periods, we limit our estimates of the probability of being exposed to two separate distinct time intervals: 1980–1989 and 1990–1996. Through the entire period, an exposed individual might have haphazardly consumed contaminated beef at least once, with the probability of being exposed depending on the exposure intensity of the infectious agent within each of the two time periods. Specifically, suppose that a certain period 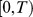 can be divided into disjoint time intervals 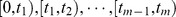. Let 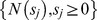 be the number of times an individual consumed a meat product containing BSE infectivity or was contaminated from an infected bovine slaughtered for consumption during the time interval 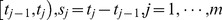. Because this is a rare event, the count 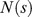 is in essence a Poisson process with intensity 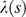 of an event’s occurrence within a time unit. Suppose that the time-dependent intensity 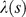 is approximately constant within each of the disjointed time intervals 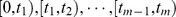. Given that the unit of time is weeks, the probability of being exposed to the BSE agent during 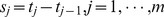 is



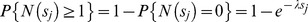
(4)


Then, following the property of a Poisson process, the probability of being exposed at least once during 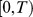 is the opposite of the probability of not being exposed through each of the disjoint time intervals 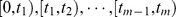, i.e.,
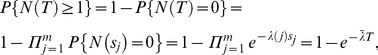
(5)where 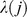 is the constant intensity within the time interval 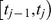, 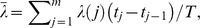 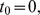 and 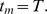 Therefore, it is sufficient to estimate the mean exposure intensities for the two periods 1980–1989 and 1990–1996, separated by the SBO legislation, for heterogeneous distributions of the infected meat products. Because of age and gender differences in beef consumption patterns [28]–[32], we estimate the mean intensities separately by gender for each of the age groups: 1.5–3, 4–10, 11–18, and 19 years old and above, with no difference between boys and girls under age 3 [28]–[32].

We use 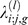 to denote the mean age group and gender-specific exposure intensity during the BSE epidemic period, where 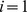 and 2 represent the periods 1980–1989 and 1990–1996, respectively; 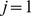 and 2 represent men and women, respectively; 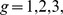 and 4 represent age groups 1.5–3, 4–10, 11–18, and 19 years old and above, and 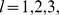 and 4 represent the type of meat products: beef and beef-containing dishes, burgers and kebabs, pies, and sausages, respectively. A reasonable intensity estimate is the frequency of consumption per week multiplied by the ratio of the total level of infectious agent (including contaminated meat) over the total amount of the meat products. Specifically, we estimate the age- and gender-specific exposure intensity of a meat product by



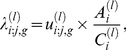
(6)where 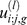 is the mean frequency of consumption per week, 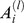 and 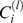 are the total amounts of SRM infectious agent and the meat products consumed within the *i*-th time period, respectively,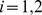; 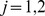; 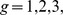 and 4; and 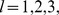 and 4. For example, the exposure intensity of an individual through the consumption of burgers is



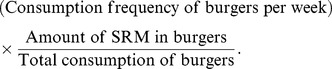
(7)The weekly consumption frequency is obtained by converting the daily ingestion quantities from Table 5.2a of NDNS [29] to a weekly estimate and dividing that estimate by the weight of a medium-sized burger, 125 g. The exposure intensities of beef and beef-containing dishes, pies, and sausages are similarly estimated, except that the unit of consumption is 60 g for sausages.

### Number of Exposed Individuals during the Period 1980 to 1996

Because of the legislation’s effects on the BSE infectious agent intensities, the birth cohorts are divided into four main categories representing different exposure patterns: pre-1970, 1970–1979, 1980–1989 and 1990–1996, due to age-dependent dietary exposures [28], [30]–[32]. The datasets of the yearly population cohorts of different age groups during the BSE epidemic period are obtained from the Office of National Statistics of the UK, together with the mortality statistic [40]. Depending on the birth cohort and the consumption of bovine meat products, an individual may have experienced differential dietary exposures during the BSE epidemic period. The probability of exposure increased with the exposure intensity and the length of time spent within the 1980–1996 period as shown in Eq. (4).

Denote by 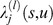 the exposure intensity to the *l*-th type of bovine meat products for an individual of gender *j* who was aged *u* at time *s*, where 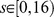 corresponds to the 1980–1996 period. Then, for an individual aged *u* who belonged to the *g*-th age category at time *s*, we have 
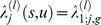
 if 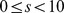; and 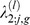 if 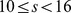. For the birth cohort of pre-1980 that went through the entire BSE epidemic period, the number of individuals of gender *j* and age *a* on January 1, 1980 who were exposed to the *l*-th type of bovine meat product is estimated to be



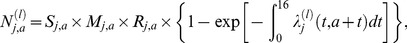
(8)where 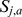, 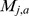, and 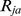 are the corresponding survival rate until the end of 2013, the total number of the individuals, and the proportion of beef consumers, respectively. For the birth cohorts 1980–1989 and 1990–1996 with a partial exposure history during the period, the number of individuals of gender *j* and age *b* on January 1, 1996 who were exposed to the *l*-th type of bovine meat product is estimated to be



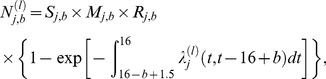
(9)where 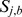, 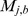, and 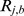 are the corresponding survival rate until the end of 2013, the total number of the individuals, and the proportion of beef consumers, respectively. Let the probabilities of exposure through dietary intake of beef and beef-containing dishes, burgers and kebabs, pies, and sausages for gender *j* and age *a* on January 1, 1980 be 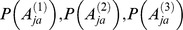, and 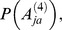 respectively. Assuming that the probabilities are mutually independent, the probability that an individual of gender *j* and age *a* who was exposed to either one of the meat products is



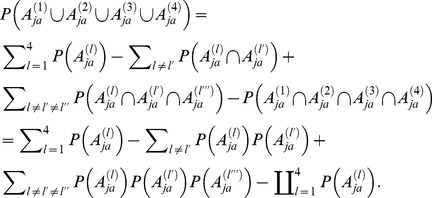
(10)


Thus, for the pre-1980 birth cohort, the number of individuals of gender *j* and age *a* who were exposed to either of the bovine meat products is



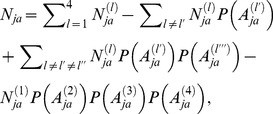
(11)where 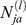 is the number of individuals of gender *j* and age *a* who were exposed to the *l*-th meat product, 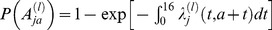. Similarly, for the birth cohorts 1980–1989 and 1990–1996, the number of individuals 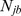 of gender *j* and age *b* who were exposed to either of the bovine meat products is calculated as (11), with 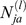 and 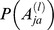 replaced by 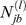 and 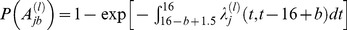. The number of exposed individuals in each of the birth cohorts – pre-1970, 1970–1979, 1980–1989 and 1990–1996 – are then obtained by summing the number of the exposed individuals whose ages *a* or *b* were in the corresponding birth cohort.

### Estimation of the Number of Subclinical Carriers and the Exposure Threshold of Developing into a vCJD Case

It has been well recognized that a species barrier must exists [4], [39], [50] when comparing the large number of individuals who might have been exposed with the only 177 vCJD cases observed to date [1]. The hypothesis that these vCJD cases might have been exposed to a rare high infectious dose also supports this observation. Alternately, the recent bioassay survey outcome suggests that a prevalence of approximately 1 in 2000, including all genotypes at PRNP codon 129, may be a subclinical carrier of the abnormal prion in the UK [13]. These individuals, though they may not develop into a vCJD case in their lifetime, may pose a threat of secondary transmission via blood transfusion. We further estimate the number of subclinical carriers making use of the survey findings and the estimated number of exposed individuals from above. Also, we adopt the threshold hypothesis [4], [34]–[39] that an individual must consume a sufficiently high infectious dose to trigger exponential growth in the brain [35], and we estimate the threshold dose by fitting an extreme-value distribution.

Let the mean infection susceptibility for the birth cohort group *g* of the G genotype at PRNP codon 129 be 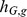. To estimate 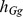, we assign a prior distribution to the susceptibility estimate of Valleron et al. [17] for each birth cohort (the force of infection at age 50, 20, 10, and 3 for the birth cohorts pre-1970, 1970–1979, 1980–1989 and 1990–1996, respectively, was adopted for the mean of the corresponding prior distribution) and incorporate the survey outcomes of Gill et al. [13] to obtain a posterior estimate. A binomial distribution with a sample size including the estimated number of exposed individuals and the parameter 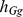 is fitted using an MCMC simulation procedure. Because the prevalence survey includes all participants regardless of whether they were beef consumers or were exposed, we calculate the specified prevalence 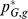 for those who are of the g birth cohort and genotype G, and who are beef consumers and have been exposed according to the following equation:



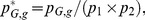
(12)where 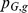 is the reported prevalence for the g birth cohort of genotype G [13], 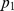 is the percentage of beef consumers for the g birth cohort [29], and 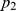 is the estimated probability of being exposed (the ratio of the estimated number of exposed individuals over the number of beef consumers in that birth cohort). We obtain the number of samples of genotype G surveyed by multiplying the total number surveyed by the corresponding genotype proportion for each birth cohort. Because the susceptibility estimate of Valleron et al. [17] is for the genotype MM, the posterior susceptibility for the MV and VV genotype is mainly based on the prevalence of Gill et al. [13] adjusted following Eq. (12). In contrast, for the birth cohort 1990–1996 not involved in the survey, the susceptibility is mainly based on the estimate from Valleron et al. [17].

We estimate the threshold dose based on an extreme-value distribution model fitted for the exposure dose. We assume that the threshold dose is *c*, irrespective of different age and gender. Because all 174 vCJD cases (excluding 3 cases via blood transfusion) are of MM genotype, we estimate the threshold only based on the number of exposed individuals of this genotype. To obtain the exposure distribution, we multiply the estimated total infectious dose, in units of bID_50_, consumed over the entire 1980–1996 period [16], [31], [32], [42] by the ratio of bovine meat consumption of each of the birth cohorts pre-1970, 1970–1979, 1980–1989 and 1990–1996. Assuming that the observed vCJD cases are those that were exposed to a very high infectious dose [4], we fit two alternative extreme-value distributions separately for the exposure dose of the exposed individuals: Weibull (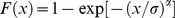) and Frechet (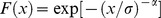). The observed vCJD cases then have a binomial distribution, with its population size being the number of exposed individuals in each birth cohort and the probability of exposure exceeding the threshold 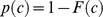 from the distributional fitting. An MCMC simulation procedure is employed to obtain the model parameters and the threshold estimates.

**Figure 1:**
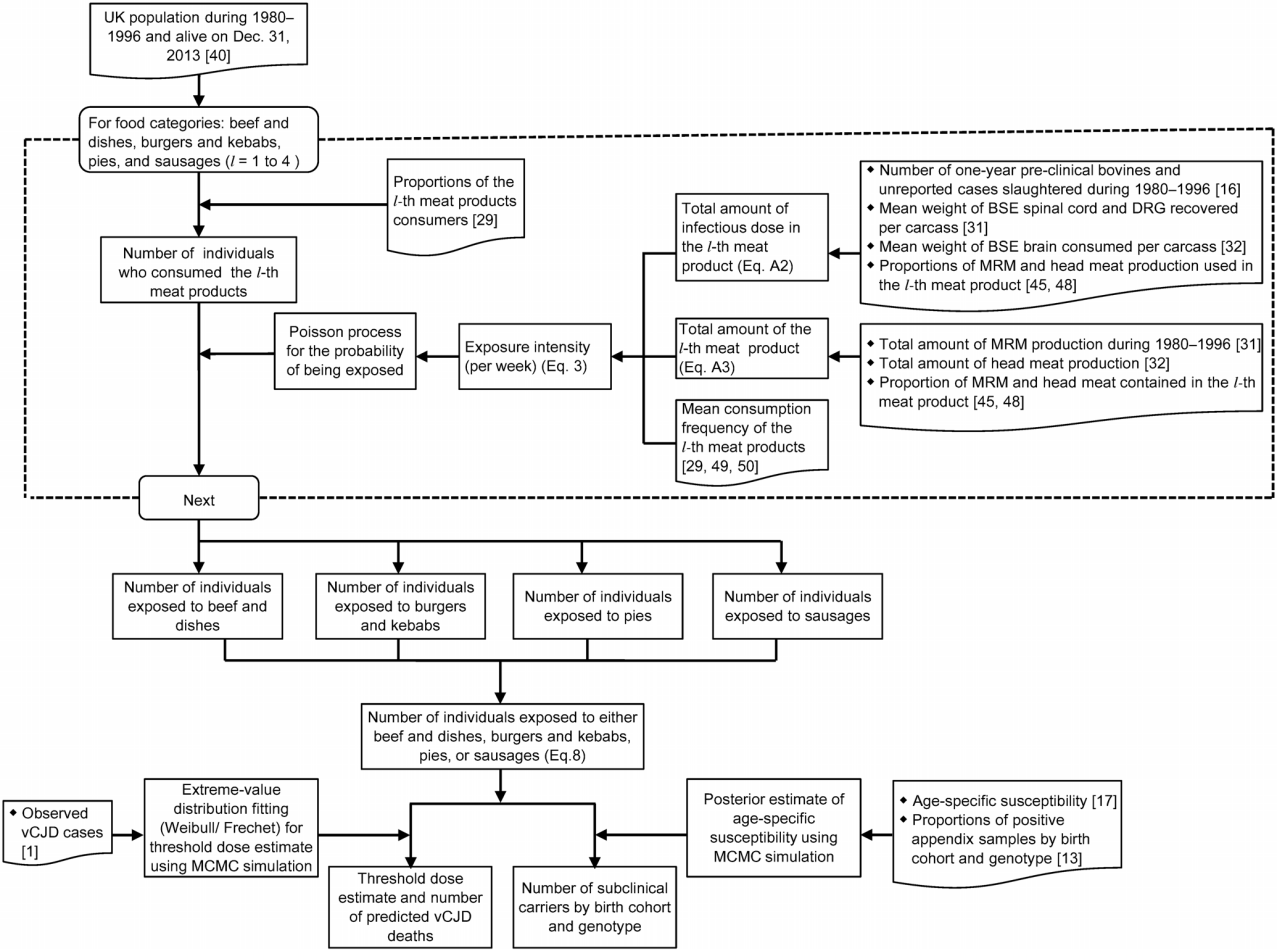
Flow chart of the estimation procedure.

For better illustration, we summarize the overall estimation procedure in the flow chart presented in Figure 1, together with the data sources we adopt in each of the steps.

## Results

### Estimates of BSE Infectious Agent Content in Meat Stocks and Exposure Intensities

**Figure 2:**
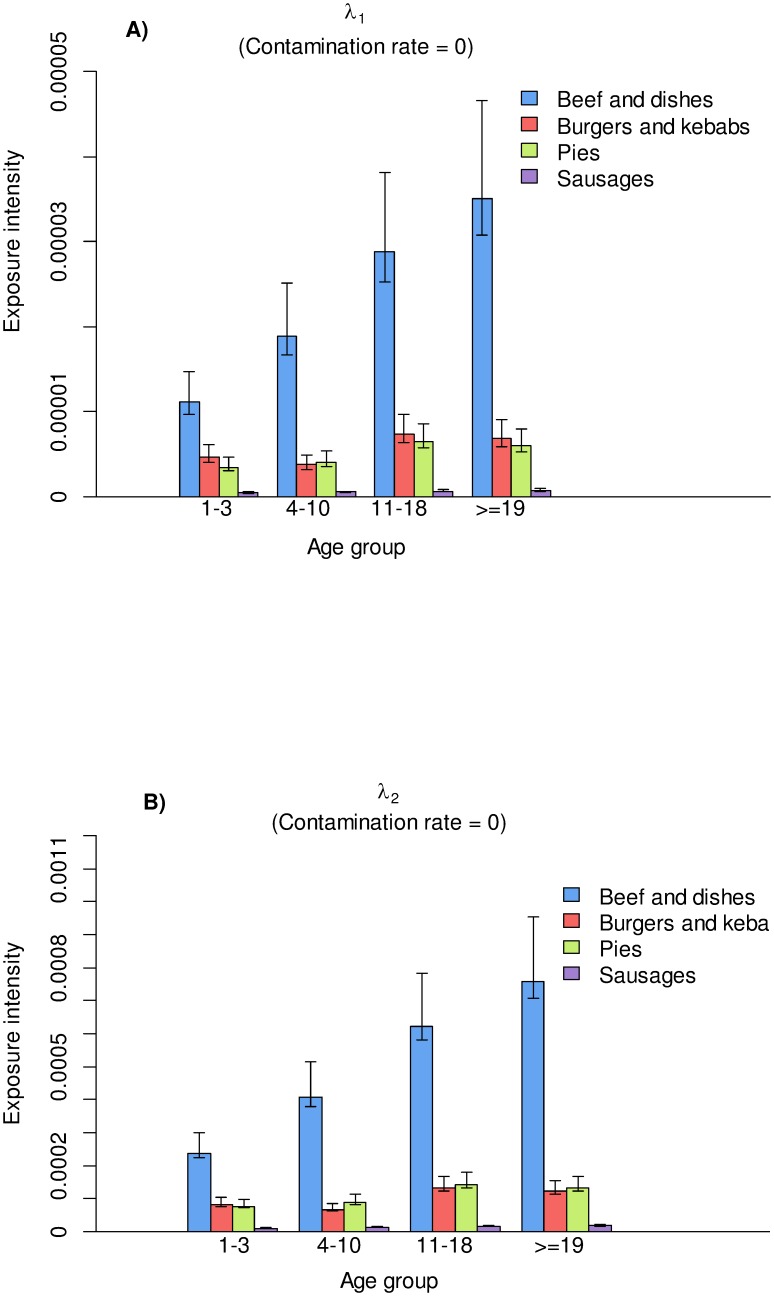
Intensities of the BSE infectious agent through dietary exposure to beef and dishes, burgers and kebabs, pies, and sausages in the age groups 1–3, 4–10, 11–18, and 19+ during the two periods. A) Intensities of λ_1_ during 1980–1989, and B) intensities of λ_2_ during 1990–1996.

Following Table 1, the estimated amount of infectious agent in the spinal cord after the SBO ban in November 1989 is less than half that of the pre-ban estimate, and the amount in the brain decreased slightly after June 1990. However, the estimated amount of DRG remains the same throughout the different periods. We calculate the exposure intensities of Eq. (5) for each of the food categories across the two major periods 1980–1989 and 1990–1996 and stratify by gender and age group. Figure 2 shows the mean intensities of dietary exposure to beef and beef-containing dishes, burgers and kebabs, pies, and sausages of different age groups –1–3, 4–10, 11–18, and 19+ years old – in the two periods. The intensity of dietary exposure to beef and beef-containing dishes is substantially higher than the intensities of exposure to other meat products across the age groups. This difference is mainly due to the higher percentages of beef and beef-containing dishes consumers in the UK (approximately 3 times the percentages of burger consumers for different gender and age categories), as well as the higher consumption frequencies [29]. The exposure intensity in beef and beef-containing dishes increases with age, with the highest exposure intensities in the adult group (19+). The intensities in burgers and kebabs, pies, and sausages are approximately the same across different age groups and periods, with the lowest intensities found for sausages. Because of the higher estimated number of one-year pre-clinical bovines slaughtered for consumption, despite lower MRM production in the 1990–1996 period [15], [16], [31], the simulated intensities in this period are approximately one order higher than those in the 1980–1989 period for all the age groups and food categories.

### Estimated Number of Exposed and Subclinical Individuals in Each Birth Cohort of All Genotypes

**Figure 3:**
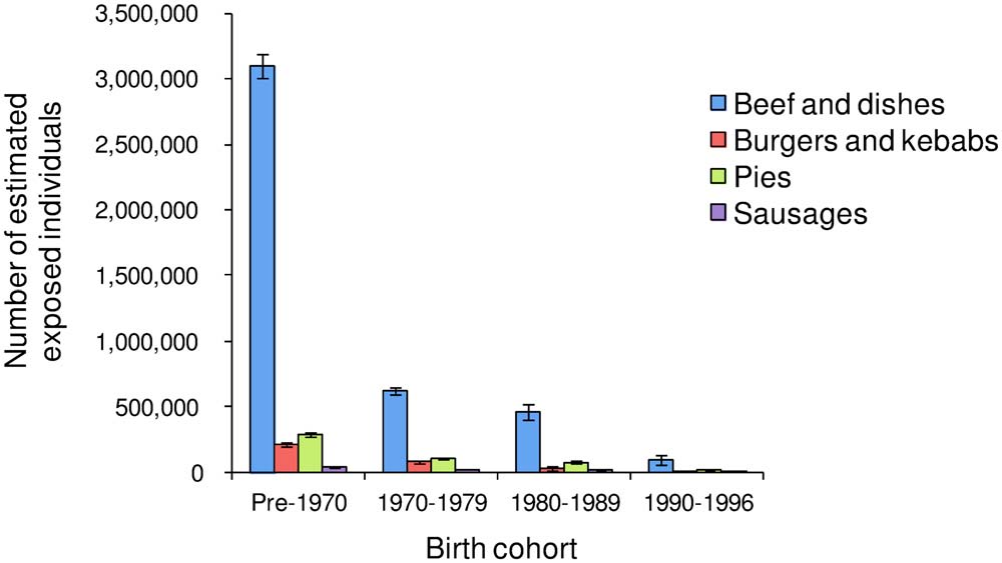
Estimated numbers of exposed individuals through dietary intake of beef and dishes, burgers and kebabs, pies, and sausages by the birth cohorts of pre-1970, 1970–1979, 1980–1989, and 1990–1996 during the period 1980 to 1996.

Figure 3 displays the estimated number of individuals exposed to beef and beef-containing dishes, burgers and kebabs, pies, and sausages for each of the birth cohorts: pre-1970, 1970–1979, 1980–1989, and 1990–1996, during the period 1980 to 1996. Most of the individuals were exposed through dietary intake of beef and beef-containing dishes: more than 3,000,000 individuals in the pre-1970 birth cohort, followed by slightly more than 500,000 in the 1970–1979 cohort. Estimates of the number of individuals exposed to burgers and kebabs, pies, and sausages are much less than estimates of the number exposed to beef and beef-containing dishes. The number of individuals exposed through intake of pies is slightly higher than the number exposed through burgers and kebabs. Only a small number of individuals was estimated to be exposed through intake of sausages.

Using Eq. (11), Table 3 lists the estimated number of exposed individuals for each of the birth cohorts, pre-1970, 1970–1979, 1980–1989, and 1990–1996, during the period 1980 to 1996, together with a 95% confidence interval (CI) based on the simulated range of the exposure intensities for the extent of uncertainty. The estimated total number is 5,089,027 (95% CI 4,514,963–6,410,317), with approximately 70% in the pre-1970 birth cohort. However, the percentage of observed vCJD cases in this cohort is only 37% ( = 65/174). In contrast, the estimated proportions of exposed individuals in the 1970–1979 and 1980–1989 birth cohorts are 16% and 11%, whereas the corresponding observed vCJD cases are 69 (40%) and 40 (23%), respectively. The excessive estimated number of exposed individuals in the older birth cohort supports the theory that younger individuals are much more susceptible to developing into a vCJD case [17]. However, only 2% (119,765, 95% CI 104,908–157,050) of the estimated exposed individuals are in the 1990–1996 birth cohort, with no vCJD incident observed in this cohort. For the estimated number of subclinical carriers, the total numbers for the MM, MV, and VV genotypes are 13,660, 10,079, and 8,116, with 95% CIs 11,641–18,061, 8,186–13,985, and 7,022–10,495, respectively. The total estimated number of all genotypes is 31,855, which is very close to the estimated number of 31,405 from Gill et al. [13] (493 per million, current population size in UK is approximately 63.7 million). The estimated subclinical prevalence for the pre-1970, 1980–1989, and 1990–1996 cohorts of the MM genotype are 5260, 2660, 2431, and 3309, respectively. Because no positive outcomes are found for some of the birth cohorts (1980–1989 for MV, and 1970–1979 and 1980–1989 for VV) [13] and very small posterior susceptibility estimates, the estimated number for these categories is 0.

To further justify the threshold hypothesis [4], [34]–[39], in Table 3 we list the parameter estimates of the extreme-value Weibull and Frechet distributions, comparing the estimated exposed individuals with the observed vCJD incidents, together with the means and threshold exposure dose estimates of each birth cohort. The mean exposed dose ranges from 0.26 bID_50_ to 0.73 bID_50_, with a threshold estimate of 11.6 bID_50_ (95% CI 9.5–13.1 bID_50_) and 10.1 bID_50_ (95% CI 9.5–10.8 bID_50_) based on the Weibull and Frechet distribution fittings, respectively. For both the extreme-value distribution fittings, the predicted numbers of vCJD cases in each cohort very closely match the observed cases, with a closer fit of the Weibull distribution.

Table 4 lists the estimated numbers of the exposed and susceptible individuals considering the exposure scenario of CR of 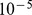 in MRM production, together with the extreme-value parameter estimates and the mean and threshold exposure dose estimates. The estimated total of exposed individuals increased to 10,280,903 (95% CI 9,666,132–11,267,838), with the total number of susceptible individuals reaching 46,482 (95% CI 39,764–55,933). The numbers of exposed and susceptible individuals are approximately 2 and 1.5 times those of the no-contamination scenario. However, the extreme-value parameter estimates and the threshold exposure dose estimate are close to those in Table 3 with CR = 0.

**Table 4:** Estimated numbers of exposed individuals and subclinical carriers, the mean exposed dose and the threshold estimates in bID_50_ for vCJD infection [43], and the expected numbers of vCJD cases exceeding the threshold dose based on the Weibull and Frechet extreme-value distribution fittings separately, assuming that the contamination rate is 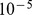.

Birth cohort	Number of exposed individuals^a^		Number of subclinical carriers		Mean exposure dose^b^	Extreme value distribution	Parameter estimates		Threshold dose estimate	Predicted vCJD cases	Observed vCJD cases^c^
		MM	MV	VV							
							α	σ			
Pre-1970	7,020,967	5,368	3,575	7,153	0.18	Weibull	1.2 (1.1–1.3)	1.8 (1.4–2.1)	12.6 (10.8–16.4)	64 (50–81)	65
	(6,603,616–7,678,196)	(5,044–5,874)	(3,358–3,913)	(6,723–7,828)		Frechet	1.6 (1.3–2.0)	0.016 (0.003–0.047)	10.0 (9.6–10.5)	66 (51–83)	
1970–1979	1,681,722	2,643	1,763	0	0.16	Weibull	1.1 (1.0–1.2)	1.7 (1.4–2.0)		69 (53–87)	69
	(1,588,184–1,837,774)	(2,493–2,892)	(1,663–1,930)	(0–0)		Frechet	1.2 (1.0–1.5)	0.009 (0.001–0.029)		71 (55–88)	
1980–1989	1,265,595	2,428	0	0	0.11	Weibull	0.6 (0.4–0.7)	0.3 (0.1–0.4)		40 (28–55)	40
	(1,185,741–1,400,092)	(2,272–2,689)	(0–0)	(0–0)		Frechet	1.1 (0.9–1.4)	0.004 (0.000–0.011)		42 (30–57)	
1990–1996	312,619	7,848	12,560	3,144	0.06	Weibull	1.7 (0.6–4.8)	0.3 (0.04–1.2)		0 (0–0)	0
	(288,591–351,776)	(6,103–10,224)	(9,690–16,455)	(2,418–4,128)		Frechet	1.2 (0.9–1.8)	0.002 (0.000–0.015)		1 (0–4)	
Total	10,280,903	18,287	17,898	10,297		Weibull				173 (131–223)	174
	(9,666,132–11,267,838)	(15,912–21,679)	(14,711–22,298)	(9,141–11,956)		Frechet				180 (136–232)	

The numbers in parentheses are the corresponding 95% confidence intervals.

a The number of exposed individuals are for all genotypes who were alive at the end of 2013.

b The mean exposure dose is obtained by dividing the estimated ratio of consumption of total amount of infectious dose in bID_50_ for each birth cohort by the corresponding number of exposed individuals who were alive at the end of 1996.

c The three cases infected through blood transfusion are excluded [1].

## Discussion

In this study, we estimated the number of exposed individuals in the UK during the BSE epidemic period 1980–1996 based on the estimated BSE-infected cattle in the last year of incubation and unreported or differentially slaughtered for consumption [15], [17], [42], the average age-specific bovine meat intake [28]–[32], and the national statistics [40]. We then estimated the numbers of subclinical carriers of abnormal prion for different genotypes at PRNP codon 129 after being exposed from the posterior susceptibility estimate with prior information obtained from the literature [13], [17]. We describe the probability of being exposed via dietary intake through the entire period by a stochastic Poisson process. This approach requires only an estimation of the mean exposure intensity of the infectious agent in bovine meat products. Thus, the assumption of the incubation period distribution and time of infection based on the observed vCJD cases, as in the back-calculation method [15]–[23] and other simulation-based approaches [24]–[27], is avoided, which significantly reduces estimation uncertainty. Furthermore, the possibility of repeated exposure [41] and the data concerning age-specific bovine meat consumption [28]–[32] are naturally taken into account in the estimation procedure. Therefore, the results provide important estimates of the prevalence of subclinical infection from mathematical modeling, in addition to the scale of exposure of the UK population to the BSE infectious agent, which cannot be derived simply from the observed vCJD cases.

Observed cases of vCJD occurring via the primary infection route of bovine meat consumption remain very small in recent years [1], as does prediction for future incidents [2]. However, uncertainty regarding the secondary infection route – blood transfusion from asymptomatic infected donors – has raised great concerns for public health and related administration strategies [3]–[7]. The estimate of approximately 32,000 exposed individuals who are potential subclinical carriers of abnormal prions provides a more concise estimate and is consistent with the results obtained from several large-scale biomarker studies on infection prevalence in the UK [5]–[13]. We obtained our estimates mainly based on the survey outcomes of Gill et al. [13], especially for the MV and VV genotypes, because previous surveys did not provide prevalence information on age and genotype [9]–[11]. Also, the infection function given by Valleron et al. [17] is for the MM genotype only. The approximately the same scale across different age groups essentially shows that both the susceptibility estimate of Valleron et al. [17] and the survey outcomes of Gill et al. [13] are reflected in the posterior estimate. A similar explanation applies to estimates for the MV and VV genotypes. These carriers are most likely subclinical to vCJD without developing into a case if they were exposed to a relatively low infectious dose. However, for the null prevalence of certain age categories and the 1990–1996 birth cohort (mainly from Valleron et al. [17]), the numbers may change substantially if positive sample(s) were detected for these categories in future surveys.

We have further justified the threshold hypothesis [4], [34]–[39] and provided an explicit threshold estimate of the infectious dose by fitting an extreme-value distribution model to the estimated number of exposed individuals and comparing that with the number of vCJD cases in each birth cohort. The existence of a threshold dose for infection has been conjectured and assessed in the literature [4], [36]–[39]. Based on the dose-response curve observed in mice, Fryer and McLean conclude that there is no evidence of the existence of such a threshold [38]. However, if this were the case in humans, the number of vCJD cases would have been far more than what has been observed to date, given our exposed individual estimate and the exponential growth rate of abnormal prions in the brain once infected [35]. The close model fitting to the observed vCJD cases justifies the threshold hypothesis. Furthermore, the threshold dose estimate of approximately 12 bID_50_ with an equivalent weight of 1.2 g of a BSE-infected bovine brain [39] also appears reasonable, which may alternatively be interpreted as the species barrier between bovine and human [39], [50].

The estimated number of exposed individuals is based on the estimation of the BSE-infected bovines in the last year of incubation and unreported or differentially slaughtered for consumption during the 1980–1996 period [16]. The figure could be much higher if all of the pre-clinical bovines and contaminated meat products made from beef that entered the food chain are considered when deriving the exposure intensity. Also, we exclude trigeminal ganglia, ileum, tonsil, spleen and eyes in our estimate of contaminated MRM because these parts are typically removed before meat consumption. However, bovine intestine was used for the manufacture of natural sausage casings prior to the SBO ban in 1989 [42]. Therefore, it is possible that individuals might be exposed through consumption of sausages with castings from contaminated intestine, which may substantially increase the number of exposed individuals. Because of the thinness, the infectivity in casings (if there is any) would be very low compared to that in contaminated MRM and head meat. Offals such as rectums and small intestines are also reported being exported to Germany for sausage manufacture and casings [45]. Based on these considerations, we choose to ignore the number of exposed individuals through this route. We rule out the possibility of being exposed by consumption of brain from preclinical BSE bovine directly, given that the major sources entering the food chain in the period were MRM and head meat [45] and none of the vCJD cases have reported eating bovine brain [42].

We adopt a Bayesian simulation approach to handle the great uncertainties in the proportions of MRM and head meat used in producing beef and beef-containing dishes, burgers and kebabs, pies, and sausages that might have contained BSE infectious agents during the 1980–1996 period. The results show that although the simulated 95% CIs cover a wide range, the estimated numbers are of approximately the same scale. Also, although the excess numbers of estimated individuals exposed due to ingestion of contaminated meat are very large, the amount of the exposure dose may be negligible for most people, except for the subclinical carriers who might be exposed to a certain amount of infectivity. As shown in Table 4, the numbers of possibly exposed individuals and subclinical carriers increase substantially with a CR of 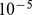. However, the threshold dose estimate remains approximately the same when the mean exposure dose decreases to about a quarter of that given in the scenario of CR = 0. Therefore, the future vCJD prediction is not expected to change because of exposure uncertainty.

In summary, the estimated current numbers of exposed individuals and those who are susceptible or carry the vCJD infectious agent may provide necessary information regarding the extent of the potential public health threat in the tail of the vCJD epidemic in the UK. The number of susceptible exposed individuals is especially important for assessing the risk of secondary transmission via blood transfusion, plasma products, or contaminated surgical instruments; assessment of this risk has been inconclusive or inconsistent based on the results of several large-scale biomarker studies [5]–[14]. Furthermore, the almost exact match between the predicted and observed vCJD cases and the threshold infectious dose estimate has greatly reduced the uncertainty regarding future incidents via the primary transmission route, food intake. However, the results obtained cannot infer the likelihood of secondary transmission from the asymptotic carriers of prion disease.
